# Supplementary figures and images for: Single-fly genome assemblies fill major phylogenomic gaps across the Drosophilidae Tree of Life
Source: PLoS Biol. 2024 Jul 18;22(7):e3002697. doi: 10.1371/journal.pbio.3002697 (PMC11257246; doi:10.1371/journal.pbio.3002697)

Tree scale: 1

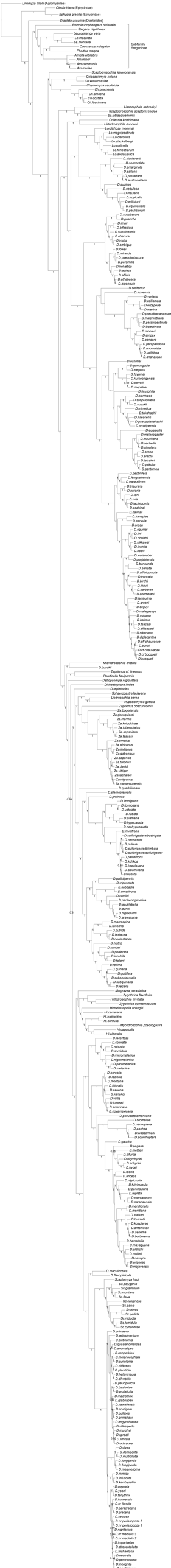

Supplement: S1 Fig — Branch lengths are scaled by the substitution rate at 4-fold degenerate sites. Node values are posterior probabilities computed by ASTRAL. The data underlying this figure can be found in https://doi.org/10.5281/zenodo.11200891. (PDF) [file pbio.3002697.s009.pdf]

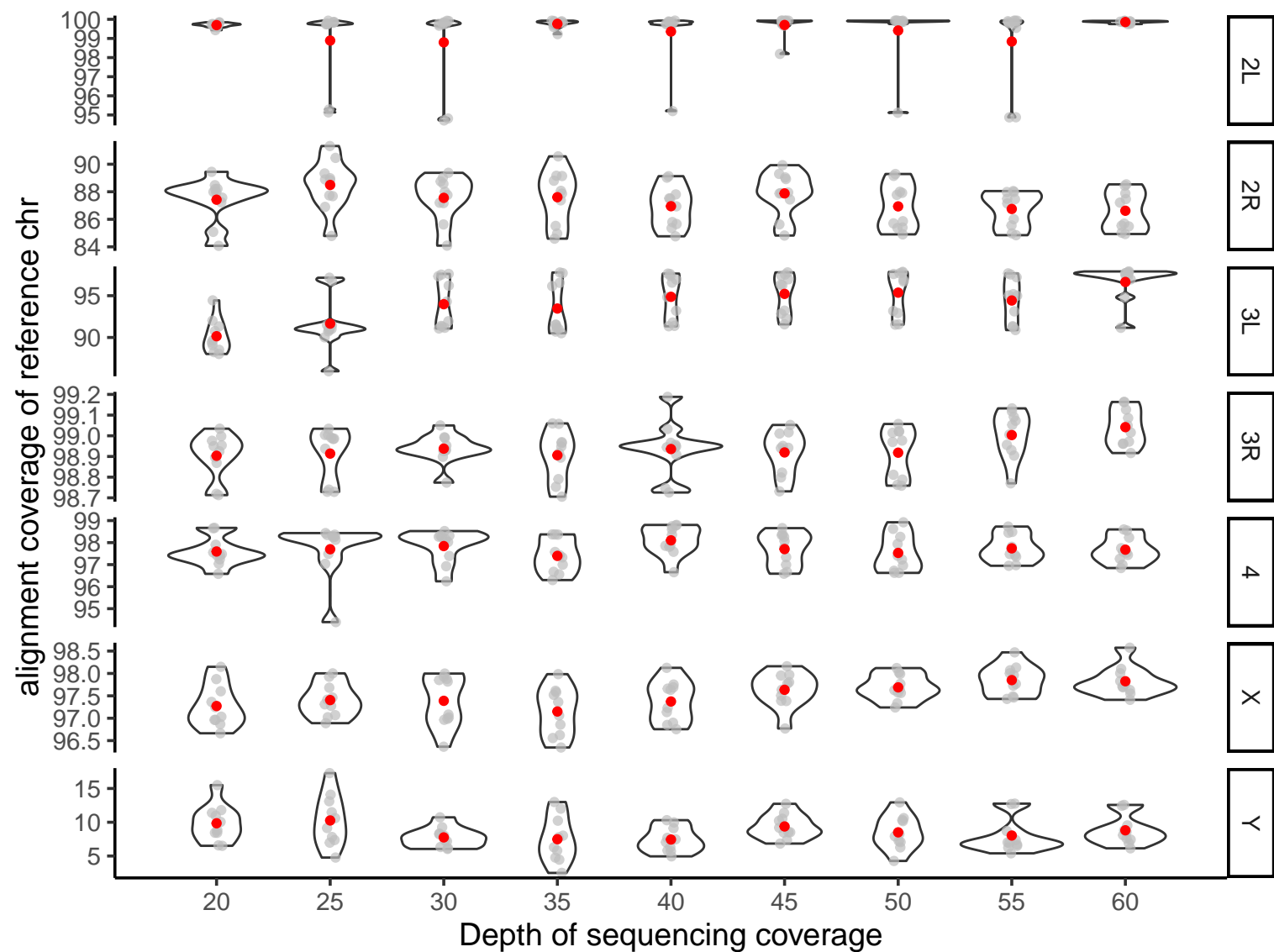

Supplement: S2 Fig — Each gray dot represents the proportion of each major chromosome of the dm6 D. melanogaster reference assembly that aligns to a de novo D. melanogaster assembly. Each dataset was generated by downsampling the original 378× ONT dataset to lower coverage. Ten replicate genomes were assembled for each coverage depth. Red dots indicate the mean across 10 replicates. The data underlying this figure can be found in https://doi.org/10.5281/zenodo.11200891. (PDF) [file pbio.3002697.s010.pdf]
