# Supplementary material for: Single-fly genome assemblies fill major phylogenomic gaps across the Drosophilidae Tree of Life
Source: PLoS Biol. 2024 Jul 18;22(7):e3002697. doi: 10.1371/journal.pbio.3002697 (PMC11257246; doi:10.1371/journal.pbio.3002697)
Supplement: Attachment — Submitted filename: response_to_reviewers.pdf [file pbio.3002697.s011.pdf]

RESPONSE TO REVIEWERS

We thank the editor(s) and reviewers for their consideration of and positive feedback on the manuscript. We describe below how we have addressed the questions, concerns, and requests brought up in the review and believe the manuscript is much improved.

Reviewer comments are highlighted in blue; our responses are in black.

Note that we do not require all raw data. Rather, we ask that all individual quantitative observations that underlie the data summarized in the figures and results of your paper be made available in one of the following forms:

> 1) Supplementary files (e.g., excel). Please ensure that all data files are uploaded as 'Supporting Information' and are invariably referred to (in the manuscript, figure legends, and the Description field when uploading your files) using the following format verbatim: S1 Data, S2 Data, etc. Multiple panels of a single or even several figures can be included as multiple sheets in one excel file that is saved using exactly the following convention: S1_Data.xlsx (using an underscore).

IMPORTANT:

a) For our wider readership, please include the word "genome" in your title, i.e. "Single-fly genome assemblies fill major phylogenomic gaps across the Drosophilidae Tree of Life"

The title has been edited.

b) Please address my Data Policy requests below; specifically, we need you to supply the numerical values underlying Figs 1 (treefile), 2, 3, 4, 5, S1 (treefile), either as a supplementary data file or as a permanent DOI'd deposition.

The data underlying the figures have been deposited on Zenodo, in accordance with the Data Policy requests: https://doi.org/10.5281/zenodo.11200891

- Figure 1: 4d_full.treefile (note: tree was plotted as a cladogram and key groups collapsed on iToL; the treefile was not modified.)
- Figure 2: S2_data.csv
- Figure 3: S3_data.csv
- Figure 4: Table_S4 (in supplementary_tables.xlsx)
- Figure 5: S5_data.csv
- Figure S1: 4d_full.treefile
- Figure S2: S6_data.csv

In addition, we uploaded several new additional files/archives in response to the reviewer feedback.

- illumina_only_assms.tar.gz: Archive of Illumina-only assemblies (FASTA) based on data downloaded off of NCBI. Assemblies generated from our own short-read data have been submitted to NCBI GenBank.
- illumina_vcfs.tar.gz: Illumina-based variant calls and BED tracks of masked bases.
- genomes.tar.gz: Genome files, for archival purposes.
- repeatModeler-lib.tar.gz: RepeatModeler2 libraries.
- diploid_genomes.tar.gz: diploid genomes and BED tracks of phased regions.

REVIEWERS' COMMENTS:

Reviewer #1:

Review of "Single-fly assemblies fill major phylogenomic gaps across the Drosophilidae Tree of Life"

This was a very interesting paper combining new lab techniques, cutting-edge genomic technologies, and useful genomic data. I really enjoyed reading it, and hope to see it published soon. I had only relatively minor requests for edits or clarifications:

-Please provide a cost breakdown for the $150/sample costs quoted here. I cannot figure out where this comes from. I'm sure the number is low, but I cannot see how it is this low given current ONT flow-cell costs.

Thank you for bringing up this point. The cost of sequencing is one of the main factors limiting the scalability of our approaches to entire clades. We were hoping to give readers a sense of the sequencing cost of the work, while highlighting the improvements in comparison to our previous (Kim et al. 2021, *eLife*) paper. It is important to note these are internal cost estimates based on the purchasing systems or sequencing providers available to us. As such, a reader planning their own sequencing experiment(s) may end up with a very different per-sample cost. Also note we reuse flow cells after digesting the old library with the ONT wash kit, allowing us to run ~8-10 samples per PromethION flow cell.

In the revised text, we clarify this these points:

[**Materials and methods**, Genomic DNA extraction and library prep]
“Flow cells were washed in between sequencing runs with the ONT EXP-WSH004 flow cell wash kit, following the manufacturer’s instructions.”

[**Materials and methods**, Sequencing cost estimate]
“The cost of sequencing is one of the major limiting factors for large genome assembly projects like this study. Here, we have highlighted an estimated sequencing cost of USD $150 per sample as a benchmark that reflects both improvements in protocols and the sequencing

technology. The specifics of this estimate are provided in **Table S6**. Note that the per-sample costs may differ from this estimate based on available sequencing resources and the scale of the project."

We also provide a new **Supplementary Table 6** for better transparency around the cost breakdown.

-Why does haploid assembly have more base-calling errors than diploid one? Won't a haploid assembly just pick one of the two alleles in a diploid? Doesn't a higher error rate imply it has to pick a third (incorrect) allele?

The reviewer is indeed correct that a haploid assembler will usually pick one of the two alleles from a diploid. While the chosen allele is thus a real variant segregating in the individual and therefore the population, a switch error will produce combinations of variants (i.e. haplotypes) not found in nature. When these errors occur between variants in close proximity, they introduce novel k-mers, and then quality score estimates from k-mer QV estimation methods are affected.

Since reference-free QV estimation is currently the best option for evaluating accuracy in *de novo* assemblies, and since switch errors may have subtle impacts on read mapping or shift the reference sequence in an unnatural way, we reasoned that we should always employ haplotype-aware assembly methods for genome assemblies of single insects (i.e. where these methods are appropriate).

To illustrate these points, we manually curated an example genome (*D. mimica*) for examples of switch errors that affect k-mer based QV estimates as described above. To describe what we did briefly, we aligned both haplotypes of the *D. mimica* diploid assembly (the phased assembly) to the haploid assembly (the unphased draft assembly) and plotted them in a genome browser (IGV). If the haploid Flye assembly, or the reference genome, represents one perfectly phased haplotype, non-reference variants (colored bars and gaps) should be found exclusively on one haplotype of the diploid assembly. In other words, the presence of non-reference variants on both diploid assembly haplotypes indicates the presence of phase switch errors in the haploid assembly.

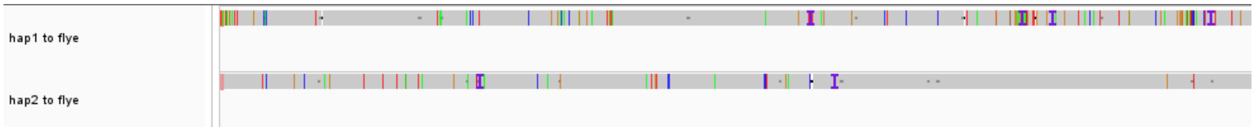

As noted above, manually inspected **individual** variants are generally well supported by both Illumina and Nanopore data. Zooming in, it is further apparent that phase switch errors link variants from opposing haplotypes in close proximity (<21 nt, the k-mer size used for QV estimation) in the unphased reference genome. In other words, phase shift errors between closely located variants introduce k-mers into the haploid assembly that would be counted as errors during reference-free QV evaluation. Three such cases are shown below.

contig_361:5,490,087-5,490,224

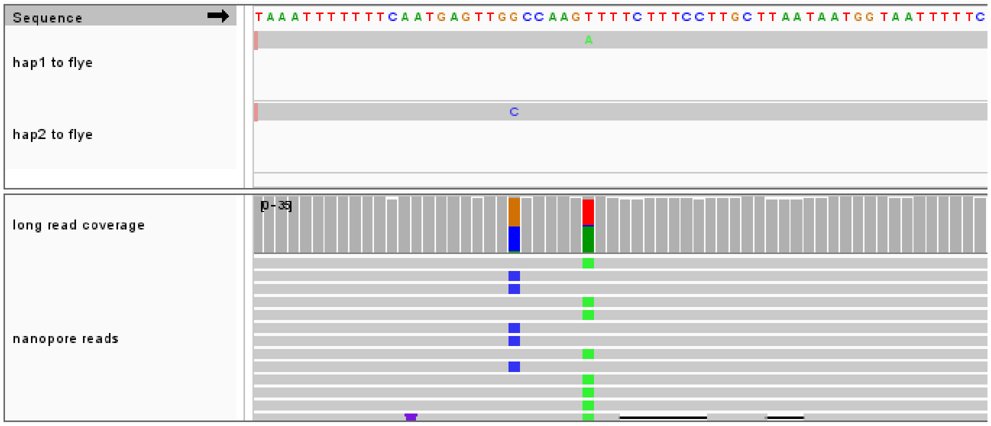

contig_361:5,478,005-5,478,279

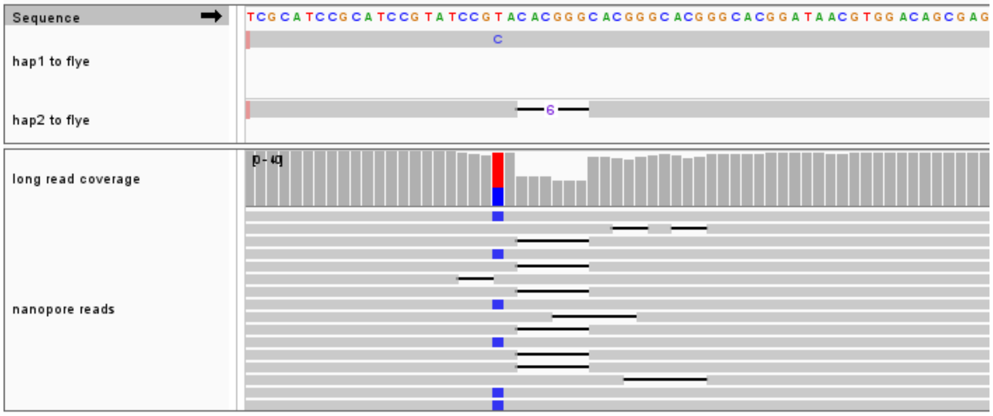

contig_361:5404822-5404959

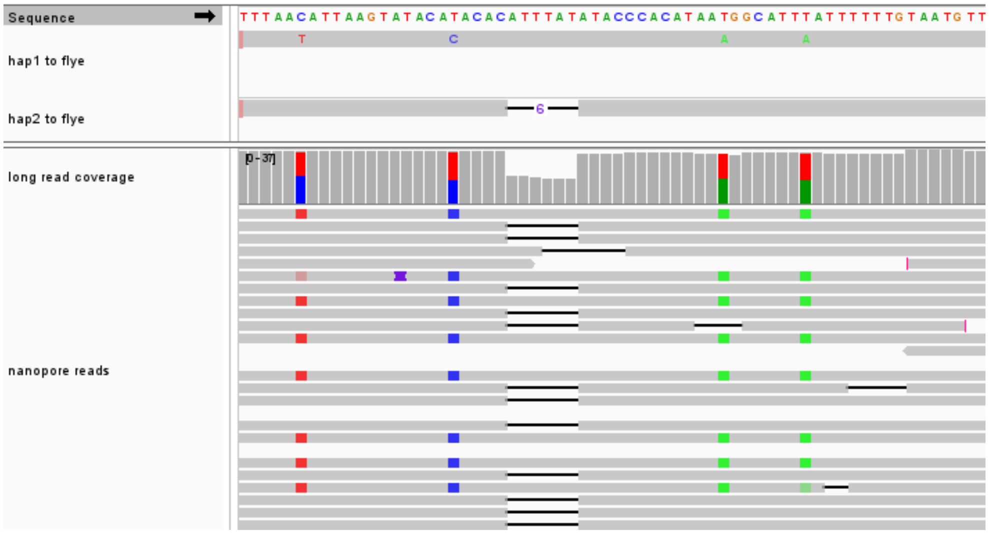

Correcting these errors by generated phased, diploid assemblies will then improve reference-free QV estimates.

In the revision, we added:

[**Results and Discussion**, Haplotype phasing improves the accuracy of single-fly assemblies]
“Further, switch errors between variants in close proximity may introduce novel k-mers into the reference genome that will be counted as errors.”

-Can you say something more about the 16 samples that were only able to be sequenced by Illumina? (Can you also make them easier to identify?) Were these samples stored dry or in ethanol? I could not find a place where the collection status of the tissue for each species was given.

Illumina-only samples are the ones that had low gDNA yields or fragmented gDNA and were thus not suitable for Nanopore sequencing. To make this clearer, we added the following text:

[**Results and Discussion**, 183 New drosophilid whole-genome sequences]
“Limited yield and/or highly fragmented gDNA limited us to Illumina sequencing for 16 specimens. The lack of Nanopore data for these challenging specimens is noted in **Table S4**. These samples tended to be older (collected between 2008-2012, with the exception of *P. flavipennis* in 2017) and smaller flies, and were all stored long-term in absolute ethanol. For these datasets, …”

Also, were these samples used in the phylogeny? It is implied (at the bottom of p.14) that they could be used, but it wasn't clear if they actually were.

Yes, all sequenced samples were used in the phylogeny. We rephrased the text to make this clearer.

[**Materials and methods**, Species tree inference from BUSCO orthologs]
“For all genome assemblies including the ones from only short-read data… ”

-I don't see a lot of "uncertainty" in the phylogeny. At worst, there is a branch with a posterior probability of 0.8. Does this branch show evidence for introgression in the Suvorov et al. paper cited? If not, some caution may be warranted with that claim. Can you report the concordance factor for this branch, and all other branches?

-Please provide your phylogeny as a Newick-formatted string in the supplement.

The way this sentence is worded is confusing and we have revised it. We meant to refer to the few instances of where branches had a local posterior probability (LPP) of less than 0.9: the deep branch with LPP=0.8, a branch with LPP=0.4 in *Amiota*, and two other branches with LPP<0.9 in the Hawaiian *Drosophila* and *immigrans* group. We modified the wording to be more specific about this and to make clear that we have not determined the specific reasons why the gene trees are more discordant at these particular nodes. We also note that in ongoing work

that uses the full set of BUSCO genes and more genomes, the deep node (the one with LPP=0.8) is confidently resolved (LPP=1).

[**Results and Discussion**, Comparative resources based on whole-genome data]
“Interestingly, we still observe some uncertainty (local posterior probability <0.9) for a few branches in the phylogeny. The cause of increased gene tree-species tree discordance at these branches is currently unknown but will be investigated using a complete set of orthologous markers in future work.”

To further address the reviewer’s comment, we have also uploaded additional trees with full annotations, annotated with just quartet scores, as well as the individual maximum likelihood gene trees so that the ASTRAL results can be reproduced.

Newick-formatted tree files are available at:
https://github.com/flyseq/2023_drosophila_assembly/tree/main/trees

Alternatively, the tree files are archived at:
https://doi.org/10.5281/zenodo.11200891

-In the caption of Figure 1 you cite ASTRAL-MP, but in the Methods you cite (but do not name explicitly) ASTRAL-III. Please clarify which was used.

This was a mistake; we used the ASTRAL-MP software and meant to cite it. This has been fixed.

[**Materials and methods**, Species tree inference from BUSCO orthologs]
“A species tree was inferred from the gene trees with ASTRAL-MP [26].”

-p. 10 of the pdf (no page numbers were included): the sentence starting "Large section of interesting…" is long and hard to understand. Consider breaking it into two sentences?

[**Introduction**]
“As a result, large sections of interesting drosophilid biodiversity are almost entirely unstudied with modern genomic tools. This includes the Scaptomyza-Hawaiian Drosophila clade, which may be one of the best examples of an adaptive radiation in nature and contains about a fifth of the species in the family [12–14], as well as many lesser-studied species or groups that may provide important context to the currently known evolutionary history of drosophilids.”

Reviewer #2:

This paper highlights development of Oxford Nanopore and Illumina genome sequencing technologies to generate quality genome assemblies from as little as 35 ng of DNA from single flies, and applies the technologies to sequence 183 new genome assemblies for 179 species (of these 121 were from single flies). This is an important advance because of the lack of need to

be able to lab culture the flies, so the single fly genomes represent samples direct from natural populations. Data were aggregated with public domain data to generate a phylogeny for 360 drosophilid and 4 outgroup species. They performed a multi-alignment of 298 of these genomes and release it all in an open resource for the research community.

1. The authors make a convincing argument that diploid assembly performs better than a non-diploid assembly of a pool of flies, even if the sample abundance is limited to the point that the genome is not fully haplotype resolved. But this implies that much of the genomes are resolved as two haplotypes. These regions should be documented/annotated.

If a region is called with two haplotypes, then it would be important to know about the SNP calling accuracy. What was the concordance of SNPs from Nanopore vs Illumina reads? The PEPPER-Margin-DeepVariant calls from the Nanopore data could be directly contrasted to the aligned Illumina reads.

To address these concerns, we have uploaded the requested data to Zenodo: https://doi.org/10.5281/zenodo.11200891

For the appropriate (single fly) samples, we have provided archives containing:

- Dual assemblies
- Variant calls
- BED intervals of phased data

Illumina variant calls were generated and compared to ONT-based variant calls to demonstrate the concordance of the two technologies. New details are provided in **Table S4**. Variant calls for most samples were highly concordant:

[**Results and Discussion**, Haplotype phasing improves the accuracy of single-fly assemblies]
“Variants called separately with Illumina and ONT reads are in general highly (>90%) concordant for 90 out of 101 tested samples (**Table S4**), further indicating the effectiveness of even modestly long reads for variant calling and phasing here.”

The “challenging samples” with lower quality scores and lower variant call consensus are mentioned:

[**Results and Discussion**, 183 New drosophilid whole-genome sequences]
“[Factors limiting assembly quality…] reducing on-target read coverage of both Illumina and Nanopore reads and thus genome contiguity, accuracy, and variant call concordance.”

We added new Methods:

[**Materials and Methods**, Additional quality control]
“For assessment of variant call concordance between Illumina and ONT variant calls in the diploid assemblies, we restricted the comparisons to SNPs and to regions callable with Illumina

data. Sites overlapping with repetitive elements or with site-level quality scores (obtained from the gVCF) less than 20 were masked. The intersection of biallelic SNPs called separately with the Genome Analysis Toolkit 4 [69] and PEPPER-Margin-Deepvariant [65] was then computed, and per-base pair heterozygosity was estimated by dividing the number of Illumina-based SNPs by the length of unmasked sequences in each genome."

The downsampling of their 384x melanogaster genome did not get at this. Why not estimate pi from each genome?

We now provide SNP-based estimates of pi (hets/bp) using Illumina data for each single-fly assembly in **Table S4**. These estimates were not computed for the assemblies from inbred lines since they were done with large pools of flies (total number not recorded). The extent and determinants of the variation in pi across drosophilids will be reserved for a forthcoming popgen study in the very near future.

What was the X vs autosome contrast in nucleotide diversity?

As mentioned below, we believe that more careful work is needed to properly identify the sex chromosomes and to perform these contrasts. In addition, we plan to examine population genetic features of drosophilids in greater detail in a forthcoming study.

Which was better for obtaining the best X chromosome assemblies, single males or single females?

Can anything be said about the Y contigs?

2. This reviewer would like to see more detail on the differences between dm6 and their 384x assembly of Dmel. Just giving BUSCO and contig size is pretty limiting. What were the regions of the genome with the biggest discrepancy? Which required the greatest read depth to resolve accurately?

We will respond to these questions together.

We are cautious about evaluating sex chromosome-specific assembly outcomes for non-model species, as we do not have the data (sequencing a male and female separately) that would allow us to systematically identify sex-linked contigs in the new assemblies, particularly in the more fragmented genomes. However, we have generally noticed that the major euchromatic chromosome arms, including the X, are well assembled even at lower sequencing coverage (20× genome-wide). On the other hand, the Y chromosome is never assembled well, even at very high coverage (>60×).

To demonstrate this, we used a reference mapping based method to evaluate the proportion of each major chromosome that was assembled in our downsampled *D. melanogaster* datasets. Since most of the Y chromosome is missing from the current dm6 reference genome, we

obtained the heterochromatin-enriched *D. melanogaster* assembly from Chang and Larracuente 2019 *Genetics* to use as the mapping reference. We added the following text to describe this:

[**Results and Discussion**, Highly accurate genomes with Nanopore R10.4.1 sequencing]
"To assess which genomic regions were best and most consistently assembled, we mapped each assembly to a reference genome and computed alignment coverage over each major chromosome (Figure S2). The D. melanogaster Y chromosome (estimated to be ~40 Mbp) is composed of repeat-rich heterochromatin and is poorly assembled, even in the current dm6 reference assembly (~4 Mbp). We used an alternative, heterochromatin-enriched assembly with an additional 10.6 Mbp of Y-linked sequences [29] for these reference alignment-based assessments, reasoning that it would provide a better, albeit still limited, evaluation of the completeness of the Y chromosome."

Our analyses (**Figure S2**) suggest that completeness and consensus quality are largely insensitive to coverage, (surprisingly) even for fairly low coverage (20× autosome, 10× sex chromosome) datasets. This is both bad and good news. The challenges around assembling the Y chromosome and heterochromatin do not seem like they can be solved just by adding more data. On the other hand, the data we are generating are expected to work well for assembling most of the *Drosophila* genome.

We have added the following text discussing this:

[**Results and Discussion**, Highly accurate genomes with Nanopore R10.4.1 sequencing]
"The depth of ONT sequencing coverage had little impact on assembly completeness for the major *D. melanogaster* chromosomes (**Figure S2**), roughly following the patterns exhibited by the consensus accuracy estimates. The major euchromatic chromosome arms (2L, 2R, 3L, 3R, 4, and X) were well assembled and exhibited similar high degrees of completeness (all ~90% or above) across the entire range of downsampled coverages, even though we expected about half coverage on the sex chromosomes relative to the autosomes (e.g., 10× X/Y vs. 20× autosome) from male flies. Similarly, the Y chromosome was always poorly assembled (about 10% complete) irrespective of coverage. While this result is expected given previous efforts to assemble the Y chromosome [27,29], our results further indicate that a modest increase in read lengths (~28kb read N50 in this study versus ~14kb read N50 in [29]) and increasing sequencing coverage will not automatically improve assemblies of repeat-rich heterochromatic sequences. More optimistically, these results demonstrate the effectiveness of even modest long-read datasets for assembling the majority of the genome."

3. How was the phylogenetic tree drawn with the two haplotypes? Were heterozygous sites reported as IUPAC encoding? Or was one haplotype arbitrarily chosen?

A single haploid genome (the primary assembly) was chosen for the phylogenetic reconstruction pipeline.

[**Materials and Methods**, Species tree inference from BUSCO orthologs]

“Only the primary assembly was used if haplotype-aware methods were utilized for genome assembly.”

4. The authors go to lengths to emphasize that this paper is about a shared resource. This is great, and I applaud the authors for the early release of the data. To maximize the ease of use of the resource, I suggest inclusion of a clear table of data types that are available and their links. Reads, annotated diploid assemblies, the multi-alignment, variant calls, outputs of RepeatModeler2, annotations of haplotype confidence.

We now provide **Table S8** to point the reader to the various datasets and resources generated by this study. Note, we have not generated genome annotations yet: this is a work in progress and will be released in a separate study.

5. Full disclosure - this reviewer is far from being an expert in the phylogenetics of Drosophila, so I cannot rate the arguments about taxon sampling or technical details of phylogenetic tree construction at this scale.

Reviewer #3:

The study by Kim et al. represents a significant advancement in genome assemblies for Drosophilidae, providing a valuable resource for researchers in the field. The methodologies employed for sequencing non-culturable species are useful for future research, and the overall clarity of the paper is good. However, I have several suggestions to improve the study before publication further.

First, the presentation of Tables S1 and S4 needs to be improved. Some species only have short read accession numbers like SRR12717852, but no assembly. Can we get the assemblies somewhere?

As a general policy, we avoid uploading genomes to NCBI if they are based solely upon data that we did not generate. This mostly includes species with only Illumina data on NCBI; we have Nanopore sequenced most of them by now and will have the long-read genomes in the next big data release. However, we agree that readers might wish to use these sequences. We have uploaded them alongside other supplementary data: https://doi.org/10.5281/zenodo.11200891

There are major caveats to these genomes. We generated these assemblies with the intention to only use the sequences for phylogenetic inference. For each species, a draft genome is quickly generated from the short reads, no additional quality control is performed, and BUSCO genes are plucked out of the assemblies.

[**Materials and Methods**, Data availability]
“Illumina-only assemblies generated from publicly available datasets (i.e., not generated by this work, **Table S1**) are archived at Zenodo (DOI: dx.doi.org/10.5281/zenodo.11200891)”

There is also some redundant information between Table S1 and S4. I think the authors can merge these two tables or separate the assemblies from other studies and their studies into two tables.

We considered this point carefully and believe that Tables S1 and S4 should still remain separated, despite the redundant information. Our hope is that they can serve as stand-alone items.

The intent behind Table S1 is to present a comprehensive list of the best genome sequences available for all sequenced drosophilid species at the time this manuscript was written. We have found it challenging to keep track of the various "best genomes" amidst the torrent of new releases of genomic data and hope to provide the reader with somewhat up-to-date suggestions of the best genome to use. We plan to eventually maintain a website with a live list of current and upcoming genomes, but building this is beyond the scope of the work presented here.

Table S4 is indeed highly redundant with Table S1, but its purpose is to provide the reader with sample and sequencing information for data from this study only. Further note that in some cases we assembled two genomes of the same species or for a new strain of a species with a genome; Table S1 only points to the representative genome while S4 provides information on both.

Keeping the two tables separated also makes it easier for the reader to sort the list of genomes by key features, for example, based on whether Nanopore or Illumina data were used for the genome assemblies.

In addition, the authors mentioned that several stocks are contaminated. A dedicated supplementary table listing contaminated stocks and the authors' strategies for resolution would enhance the paper's completeness. If the authors plan to update and correct records, can they provide a link (github?) that allows readers to track? These will be helpful for people who are using the assemblies to notice the possible issues.

The phrasing of this statement was not specific enough and we apologize for the confusion.

One of the difficulties in keeping track of stock contamination is that while we do almost all of the sequencing in the Petrov Lab, we rely heavily on collaborators to maintain and collect strains. We do not release data from contaminated lines even if we are able to identify the species, because we cannot determine whether the line was originally misidentified or became contaminated in the lab. If we identify contamination in a line directly ordered from the NDSSC, it is reported to the NDSSC staff.

The contamination events we have encountered are, specifically:

- *D. pandora* was actually *D. parabipectinata* (provided by Jan Hrcek, a proper *D. pandora* line was sent later, incorrect data discarded)

- *D. pallidosa* was actually *D. melanogaster* (provided by Scott Pitnick, the correct stock was re-ordered from the NDSSC and sequenced, incorrect data discarded)
- *Scaptodrosophila lativittata* 11020-0081.00 was actually *D. melanogaster* (received directly from the NDSSC, contamination reported)
- *D. nebulosa* (14030–0761.01) was actually *D. sucinea* (from Kim et al. 2021 *eLife*, received directly from the NDSSC, contamination reported and correction issued at https://doi.org/10.7554/eLife.78579)

To make this clear, we have added the following text:

[**Materials and Methods**, Strain contamination]
"We have identified some contaminated fly stocks through the course of this work. All but one come from internal contamination events and so the correct stock was ordered and sequenced. *Scaptodrosophila lativittata* (NDSSC# 11020-0081.00) was obtained directly from the stock center and turned out to be *D. melanogaster*. Data from contaminated stocks are never used."

Second, as the authors mention, many genera are polyphyletic in Drosophilidae. The authors only stated which species they chose but did not explicitly discuss the alignment of their selected species with previous phylogenetic studies. For example, are there other studies that described four genera as sisters to Drosophila? I understand that the authors might have follow-up studies to talk about introgression or incomplete lineage-sorting, etc., but I think that it is necessary to review some of the previous phylogenic studies and state that their phylogeny is primarily consistent with previous studies here, with some exceptions like D. flavopinicola. Otherwise, judging whether the authors' assemblies can recapitulate what people found before is hard.

We have addressed this by including new text to better orient the reader with the context of the current phylogeny in a non-exhaustive manner. We now describe how whole genomes are able to recapitulate previous studies, but also their power to resolve both uncertain deep and recent evolutionary relationships in a way that is not possible with just a few loci.

The phrasing describing *Colocasiomyia, Chymomyza, Scaptodrosophila,* and *Lissocephala* as "sister to *Drosophila*" was unclear and has been revised to "that are outgroups to [...] *Drosophila*."

[**Results and Discussion**, Taxon sampling]
"Within the subfamily Drosophilinae, we sequenced 8 species from 4 genera (Colocasiomyia, Chymomyza, Scaptodrosophila, Lissocephala) that are outgroups to the large, well-studied, and paraphyletic genus Drosophila."

Next, we majorly revised the remainder of the relevant Results and Discussion section to provide more context of previous phylogenetic studies of Drosophilidae.

[**Results and Discussion**, Taxon sampling]

"We selected additional species for sequencing with the primary objective of improving the taxonomic diversity of genomes of species across the family Drosophilidae (Figure 1). The wealth of information in a diverse set of genomes will create many new opportunities for understanding the biology of drosophilids. Robust inference of historical evolutionary relationships among species and higher taxonomic groups is a key first step that lays the foundation for future study into drosophilid evolution. To date, the largest molecular phylogeny of the group is based on 17 genes from 704 species [7]. While these data are by far the most comprehensive in the number of species surveyed, many deep branches in the phylogeny and many of the exact relationships of species within species groups and sub-groups are not confidently resolved. More loci are needed to resolve the species tree, particularly in the presence of extensive introgression and incomplete lineage sorting [21]. Whole-genome sequencing, particularly long-read sequencing, makes thousands of orthologous loci immediately accessible and addresses these issues.

Sampling was conducted across the family as follows. From the TaxoDros database [22], family Drosophilidae is split into the lesser studied subfamily Steganinae, for which we sequenced 9 species from 5 genera (*Stegana, Leucophenga, Phortica, Cacoxenus, Amiota*), and the better known subfamily Drosophilinae. Within the subfamily Drosophilinae, we sequenced 8 species from 4 genera (*Colocasiomyia, Chymomyza, Scaptodrosophila, Lissocephala*) that are outgroups to the large, well-studied, and paraphyletic genus *Drosophila*. Previous studies (e.g., [7,14,23,24]) have long noted this paraphyly, but a taxonomic revision has not occurred in part due to potential effects on the nomenclature of model organisms and due to uncertainty about the placement of many taxa. We therefore sampled 22 species from 14 genera that render the genus *Drosophila* paraphyletic (*Collessia, Dettopsomyia, Dichaetophora, Hirtodrosophila, Hypselothyrea, Liodrosophila, Lordiphosa, Microdrosophila, Mulgravea, Mycodrosophila, Phorticella, Sphaerogastrella, Zygothrica, Zaprionus*).; Finally, we sampled new species from the *testacea, quinaria, robusta, melanica, repleta*, and Hawaiian *Drosophila* species groups."

Finally, we added text that describes how our phylogeny compares to the current state-of-the-art:

[**Results and Discussion**, Comparative resources based on whole-genome data]

"We inferred species relationships of these 364 genomes using 1,000 dipteran BUSCO genes [33,36] identified as complete and single copy across the most genomes (Figure S1). As expected, the relationships of the major species groups in our phylogeny tree remains mostly consistent with previous work [7,24]. The differences also reflect our much larger set of orthologs: deep-branching relationships between the clade containing *Mulgravea*, *Hirtodrosophila, Zygothrica*, and *Mycodrosophila*; *Dichaetophora*; *Dettopsomyia*; and the *Drosophila* and *Siphlodora* subgenera are confidently resolved, as well as the more recent evolutionary relationships between nearly all individual species."

As the reviewer mentioned, we are currently working on a large follow-up "tree of life" study that will use ~500 genome assemblies (~200 more than what is presented here) to build a backbone

tree and then incorporate all publicly available Sanger datasets. A more extensive discussion of the phylogenetic results is planned for that study.

I also noticed that the authors mentioned the less accurate assemblies of sex chromosomes without delving into potential reasons. It might be beneficial if they explore whether the errors stem from differences in coverage between sex chromosomes and autosomes or if the repetitive nature of sex chromosomes plays a role. Offering some insights into the location of errors could be quite enlightening.

Please see our response to Reviewer 2 regarding sex chromosome assemblies as it addresses this question.

Last, the authors mention, "The inferred ancestral drosophilid genome is 33.5 Mbp in size, about 10 Mbp larger than the sum of D. melanogaster coding sequences, and contains 97.3% of the dipteran BUSCO genes as complete and single-copy." I'm curious about the authors' interpretation of this finding.

Strictly speaking, Progressive Cactus reconstructs an ancestral genome for each node in the guide tree. The ancestral drosophilid genome will be composed of sequences that are alignable across the 298 species used to generate the Progressive Cactus alignment. It is not surprising that the dipteran BUSCO genes are present in the ancestral genome: these genes are expected to exist as single-copy orthologs across most dipteran species (most recent common ancestor ~240 MYA).

Without performing a formal analysis of sequence conservation and estimation of evolutionary rates yet, the enrichment of expected functional sequence in the ancestral genome implies that the ancestral assembly represents a core set of sequences shared amongst the drosophilids. While much of this should be coding DNA, conservation of a lesser proportion of introns and intergenic sequences is seen (**Figure 5**). The ancestral genome of 33.5 Mbp may provide an estimate of the total amount of functional sequences (coding and non-coding) that are conserved across Drosophilidae.

[**Results and Discussion**, Comparative resources based on whole-genome data]
“This suggests the ancestral assembly is enriched for functional sequences and provides an upper bound for the total amount of functional sequence conserved across Drosophilidae.”

Minor comments:

1. The authors mentioned: For some of these samples, we made several attempts at a genome assembly and presented the best one here." Can the authors say which these samples are and how they were done?

We had issues assembling genomes from mushroom feeders (*quinaria* group and *Hirtodrosophila*) and some of the older Hawaiian *Drosophila* species. In general, we

encountered two kinds of issues: contamination from compounds that interfered with sequencing and contamination from off-target microbial sequences. We believe that the former issue might arise from either diet or sample age; we have tried many different DNA purification protocols (e.g., even those designed to purify gDNA from plant material) to no effect. The latter issue – difficult to deal with because there is no easy way of determining levels of microbial contamination beforehand – is dealt with by increasing sequencing throughput or by re-doing the assembly with another specimen. The species for which we attempted multiple assemblies are: *D. subquinaria*, *D. suboccidentalis*, *D. recens*, and *D. rellima*.

[**Results and Discussion**, 183 New drosophilid whole-genome sequences]
"For some of these samples (specifically, *D. subquinaria*, *D. suboccidentalis*, *D. recens*, and *D. rellima*), we made multiple attempts at a single-fly genome assembly and present the best one here."

2. My Excel can't see the last column of Table S5

Thanks for pointing this out. This issue probably occurred while converting Google Sheets to Excel and has been fixed.

3. The authors have identified X-linked contigs to verify fly sex using Muller elements. It will be great for them to provide the data.

We hesitate to release the X-linked contigs as this analysis was, at best, a cursory sanity check that coverage over putative X-linked markers (BUSCO genes) was consistent with microscopy sexing of the sequenced flies. Specifically, this consisted of two BUSCO runs on *D. melanogaster* and a new genome and a comparison of read coverage over the identified BUSCO genes. This was not intended to be the primary method for sexing. Unfortunately, we think the presence or lack of a BUSCO gene linked to the X chromosome in *D. melanogaster* is not definitive evidence that a contig is from the X chromosome in another species. Further sequencing of separated males and females will be necessary to properly assign contigs to the X – especially in more fragmented genomes.

Other notable changes

Table S1: Genome accessions have been changed to reflect NCBI releases. Accessions for genomes for *D. pallidosa* and *Mycodrosophila poeciliogastra* remain "PENDING" as the genomes appear to still be in a processing state. Irrespective of their release state, all genomes have been submitted under the NCBI BioProject created for this study.
